# Reproductive health

**DOI:** 10.7554/eLife.102432

**Published:** 2025-01-29

**Authors:** Wei Yan

**Affiliations:** 1 eLife Sciences Publications Cambridge United Kingdom

**Keywords:** reproductive biology, reproductive health, fertility, pregnancy, reproductive disorders

## Abstract

The articles in this special issue highlight the diversity and complexity of research into reproductive health, including the need for a better understanding of the fundamental biology of reproduction and for new treatments for a range of reproductive disorders.

Reproductive health issues affect hundreds of millions of people around the world every year, shaping everything from fertility and pregnancy outcomes to broader societal concerns, including demographic trends and healthcare disparities (ACOG, 2024). However, our knowledge and understanding of human reproduction and its associated disorders is incomplete. This is partly because research into reproductive health has historically been overlooked, with funding and attention disproportionately favoring other areas of medical research (Mercuri and Cox, 2022).

The decision to launch this special issue on reproductive health was motivated by several factors. One key driver was the recognition of how little we still know about the fundamental biology of reproduction and its disorders. Compounding this gap is the pressing need for robust, evidence-based insights to inform public debate and policy on topics such as abortion and IVF. Moreover, recent breakthroughs in research – such as organoid models, multi-omics techniques, and CRISPR-mediated gene editing – have opened up unprecedented opportunities to explore longstanding questions, catalyzing fresh momentum in the field.

The articles in the special issue span a wide array of topics, reflecting the diversity and complexity of reproductive health research. Several articles delve into the neuroendocrine regulation of reproduction, exploring how the brain and endocrine system orchestrate reproductive processes (Sáenz de Miera et al., 2024; Qiu et al., 2024; Hackwell et al., 2024). Others highlight epigenetic mechanisms, offering insights into how epigenetic changes influence fertility, pregnancy, and even the health of future generations (Verdikt et al., 2023; Cincotta et al., 2024; Lehle et al., 2024). Pregnancy and placental biology is another key theme, with articles addressing the mechanisms of normal gestation as well as the causes of complications such as preeclampsia and recurrent pregnancy loss (Liao et al., 2024; Wu et al., 2024). Further contributions investigate gamete biology and fertilization, illuminating the molecular and cellular events critical to sperm and egg formation (Wang et al., 2023; Muroňová et al., 2024; Granados-Aparici et al., 2024), and to sperm-egg interactions (Elofsson et al., 2024). Other articles offer new perspectives on the decline in fertility associated with aging (Huang et al., 2024; Amir et al., 2024).

Despite these advances, significant challenges remain. A persistent issue is the lack of comprehensive data on how lifestyle (such as dietary habits and levels of physical activity) and environmental factors (such as exposure to chemicals that disrupt the endocrine system) affect reproductive health. Equally concerning are disparities in reproductive health outcomes, which disproportionately impact marginalized communities (ACOG, 2024). Moreover, the biological mechanisms underlying common reproductive disorders, such as polycystic ovary syndrome, infertility, and recurrent pregnancy loss, remain poorly understood, hampering the development of effective treatments (NASEM, 2024). Outstanding open questions include: how do interactions between genetics, epigenetics and environmental factors shape reproductive health? And do assisted reproductive technologies have any long-term impacts on offspring health? Emerging organoid systems offer great promise, but their potential to model complex reproductive disorders effectively is still being explored (Kim et al., 2020).

By bringing together a wide range of innovative studies, we hope that this special issue will spark new conversations and collaborations among researchers, clinicians and policymakers. Moreover, in addition to deepening our understanding of reproductive biology, we hope that some of work reported in these articles will pave the way for advances in clinical medicine and more equitable healthcare practices worldwide. Finally, by highlighting the outstanding scientific challenges within reproductive health research, and the potential health benefits to billions of people around the world, we hope to encourage more researchers to work in the field and to convince governments and funding agencies of the need to increase their investment in this research for the good of both science and society.
